# The Influence of Total Quality Recovery in Perceived Enjoyment during Football Specific Training

**Published:** 2018-08

**Authors:** Okba SELMI, Nejmeddine OUERGHI, Wissam BEN KHALIFA, Fedi AMARA, Anissa BOUASSIDA

**Affiliations:** 1. Research Unit, Sportive Performance and Physical Rehabilitation, High Institute of Sports and Physical Education of Kef, University of Jendouba, Kef, Tunisia; 2. Science Department of Life, Bizerte Faculty of Sciences, University of Carthage, 7021 Zarzouna, Bizerte, Tunisia

## Dear Editor-in-Chief

Football coaches frequently use small-sided games (SSGs) to concomitantly train the physical, tactical, technical and psychological components in the way similar to match play (1). The SSG is more efficient, motivating and enjoyable for the participants than other regime training (2). SSG caused greater perceived enjoyment (PE) than generic training among youth elite football players (3). Fatigue and lack of recovery negatively affect athlete performance and produces a psychometric disturbance during training and sports competitions. Indeed, recovery is an essential element to avoid fatigue (3). In this regard, the Total Quality Recovery Scale (TQR) (4), is a good predictor of the recovery state of athletes. In agreement as to TQR was strongly associated with performance and well-being of athletes (5). To the best of our knowledge, no study has examined the influence of recovery state in enjoyment in football training. The purpose of this study was to assess the effects of the TQR on PE rating during small-sided games in young football players during the Tunisian championships season in 2014–2015.

Sixteen young male football players from a national team (mean±SD: age=16.5±0.6 yr, height=178.2± 64 cm, body mass=68.4 ±5.4 kg, and body fat percentage=10.6±0.8) gave their written consent to participate in the study.

The study was conducted according to the Declaration of Helsinki and the protocol was fully approved by the local Research Ethics Committee.

Three training interventions of 4 vs. 4 SSG were performed in separate days of the competitive season (1 wk interval) on an outdoor field on a playing surface of 30 m long and 18 m wide. Moreover, the coach and the physical trainer were around the pitch to encourage the participants to perform a maximum effort during the exercises and to maintain the possession of the ball. Each player was asked to rate subjectively about (i) the recovery state during the previous 24 h. Fifteen min before each training session, their recovery score on a scale from 6 to 20 (4). Five minutes after training intervention, players rated how they feel at the moment about the session training they have just been doing using Physical Activity Enjoyment Scale (6). Pearson’s correlations were used to determine the relationships between the variables of interest.

No significant correlation found between TQR and PE (*r*=0.31*, P*>0.05).

This is the first study investigating the relationship between PE (during SSG) and the TQR noted before training session and collected in order to detect recovery state. Our results revealed that the rating of PE does not seem to be influenced by the variability of the TQR with young players.

TQR are not contributing signals to altered PE during the training of SSG. TQR are used to detect the current state of players, expressing the feeling of negative adaptations to training, and impairment of psychophysiological processing in terms of function (4). In order to improve an enjoyment among players, it is important to know what motivates them to be vigorous. Enjoyment is a key predictor of commitment and performance in players (2). However, in order to be enjoyable, the modalities of exercise players engage in evoke a high intensity with technical efficiency. For these reasons, contribution in SSG is produced greater motivation and physical enjoyment in the players than other training modalities (3).

The PE-induced by a training method might vary according to types of exercise, motivation, and encouragement of the players. PE does not seem to be affected by the variability of TQR during football-specific training among young players.
